# Integrating ex situ biomimetic extraction analyses into contaminated sediment assessment and management decisions

**DOI:** 10.1093/inteam/vjae008

**Published:** 2025-01-06

**Authors:** Thomas F Parkerton, Aaron D Redman, Daniel J Letinski, Magdalena I Rakowska, Danny D Reible

**Affiliations:** EnviSci Consulting, LLC, Austin, TX, United States; ExxonMobil Biomedical Sciences, Annandale, NJ, United States; ExxonMobil Biomedical Sciences, Annandale, NJ, United States; ENVIROSTATUS, LLC, Lubbock, TX, United States; Department of Civil, Environmental, and Construction Engineering, Texas Tech University, Lubbock, TX, United States

**Keywords:** sediments, ex situ passive sampling, biomimetic extraction, toxic units, fluorometry

## Abstract

This study evaluated a novel ex situ passive sampling biomimetic extraction (BE) method to estimate toxic potency in sediments. Gas chromatography with flame ionization detection (GC-FID) analysis of polydimethylsiloxane fibers equilibrated with field sediments was used to quantify bioavailable polyaromatic hydrocarbons (PAHs) and other unresolved, site-specific contaminant mixtures. This method is biomimetic because contaminants partition to the fiber based on hydrophobicity and abundance, and GC-FID quantification accounts for all constituents absorbed to the fiber that may contribute to toxicity. This measurement was compared with conventional approaches that rely on bulk sediment or porewater measurements of a targeted suite of PAHs. The specific objectives of the study were to (1) describe the BE method and explain measurement translation into toxic units (TUs); (2) report sediment BE data collected across 17 diverse field sites; (3) compare TUs predicted from (i) equilibrium partitioning (EqP) calculations based on sediment total organic carbon and bulk PAH chemistry, (ii) PAH porewater concentrations derived using ex situ passive sampling, and (iii) BE concentrations; and (4) discuss implications of this analysis for benthic toxicity assessment. Results showed that TUs obtained from EqP calculations were typically 10× higher than TUs derived from measured porewater PAH concentrations, indicating reduced PAH bioavailability in field sediments. Toxic units derived using the new BE method were more conservative than EqP in one-third of the sediments investigated, which was attributed to unquantified sediment contaminants, possible fiber fouling in the more contaminated sediments, and potential background interferences in less contaminated sediments. Preliminary data are also presented, showing that fluorometric analysis provides a simpler, promising alternative for estimating sediment BE concentrations. Based on this analysis, a decision-support framework is proposed using EqP and BE based TU metrics. Future research priorities are described for supporting framework implementation and extending use of BE analyses to remedial design and monitoring.

## Teaser

**Key points**

- A new ex situ biomimetic extraction (BE) method was applied to 73 field sediments from 17 sites to quantifiy the bioavailability of PAHs and other unresolved compounds in contaminated sediments. The BE results were compared to equilibrium partitioning and passive sampling PAH sediment chemistry-based toxicity metrics and integrated into a decision framework for contaminated sediment management.

## Introduction

Sediments are an integral component of aquatic ecosystem structure and function. Legacy and ongoing sources of pollutants that contaminate sediments can adversely affect the health and impair intended uses and associated ecosystem services of waterbodies (Krumins et al., 2013; Wang et al., 2021). Risk assessment is used to evaluate the environmental and human health concerns posed by contaminated sediments and determine whether remedial action is warranted. Remediation options are often costly and pose additional risks to people and the environment, so these considerations are taken into account in deciding whether a screening or higher tier risk assessment is needed to support remedy selection (Apitz et al., 2005b). Once remedies are selected to mitigate unacceptable risks, subsequent implementation must address both operational and engineering challenges (Apitz & Power, 2002; Apitz et al., 2005a).

An essential component of contaminated sediment site characterization includes an evaluation of risks posed to benthic organisms. This assessment usually includes comparing observed sediment concentrations with sediment quality guidelines (SQGs) derived for benthic life (USEPA, 2005). A recent critical review of SQGs for polyaromatic hydrocarbons (PAHs) is provided by McGrath et al., 2019. Two general approaches have been used to establish numerical criteria. The first approach relies on the association between total PAH concentrations and adverse effects observed on benthos in laboratory or field studies to establish a concentration below which adverse effects are rarely observed. The second approach relies on the combined use of equilibrium partitioning (EqP) to estimate dissolved porewater concentrations in sediments and a mechanistic effects model (i.e., target lipid model or TLM) that is calibrated using aquatic toxicity data to establish a hazard-based porewater concentration that is protective of benthic life. This approach has been extended to PAH mixtures by applying an additive toxic unit (TU) model and forms the basis for the U.S. Environmental Protection Agency (USEPA) equilibrium sediment benchmarks (ESBs) as detailed by Burgess et al. (2013). The EqP method assumes an equilibrium of chemical with food, water, organism, and the sediment (Kwok et al., 2014; McGrath et al., 2019) so that the anticipated risks can be normalized to the freely dissolved concentrations in sediment porewater irrespective of the actual exposure route. Critical evaluations of EqP assumptions have been provided elsewhere and were concluded to be acceptable for SQG derivation (ECETOC, 2020; Hiki et al., 2022).

The ESB approach provides a number of advantages for use in risk screening evaluations, including the underlying mechanistic basis of adverse effects; the ability to consider the site‐specific profile and toxicity of individual parent and alkyl PAHs, consideration of contaminant bioavailability through organic carbon normalization, and improved concordance with observed sediment toxicity data when compared with association-based SQGs (McGrath et al., 2019). Further, ESBs can also be extended to other important classes of sediment contaminants beyond PAHs, such as PCBs (Fuchsman et al., 2023). However, ESBs have two important drawbacks. First, the site-specific bioavailability of contaminants in sediments may not be reliably predicted using EqP. Specifically, the presence of soot, or black carbon, or weathered oil or other phases can increase binding to sediments, thereby reducing freely dissolved concentrations (Arp et al., 2009; Cornelissen et al., 2005; Ghosh, 2007; Koelmans et al., 2006). Further, nonequilibrium conditions can occur in field sediments (Burgess et al., 2023). As a result, ESBs are expected to be conservative, and although valuable in screening risk evaluations, can overstate the observed toxicity of field sediments (McGrath et al., 2019). In fact, heterogeneity in sediment binding phases and the corresponding variation in site-specific sediment-water partitioning and contaminant bioavailability often provides the key focus in higher tier benthic risk evaluations. Recent technical guidance recognizes this shortcoming by providing guidance for establishing site-specific porewater-based remediation goals (Burkhard & Mount, 2017). This refined methodology leverages technological advances in passive sampling methods, allowing sensitive and reliable quantification of freely dissolved sediment porewater concentrations (USEPA, 2017). An important implication is that this new guidance supports remedies that focus on reducing porewater concentrations such as use of in situ amendments and natural contaminant degradation. Such remedies may offer lower inherent implementation risks and associated costs than traditional bulk sediment removal via dredging (Kupryianchyk et al., 2015; Li et al. 2020; Patmont et al. 2015). Second, ESB are available only for a limited number of routinely measured analytes. For example, in USEPA ESBs for PAH mixtures, 34 parent and alkyl homologs are used to define “total PAH” and calculate summed TUs. However, other aromatic and aliphatic hydrocarbons that comprise the unresolved complex mixture (UCM) may contribute to observed toxicity in hydrocarbon contaminated sediments (Du et al., 2012; Scarlett et al., 2007). Techniques for quantification, risk assessment, and remediation of such hydrocarbons have been identified as an important research need (Ramadass et al., 2021). In summary, ESBs incorporate assumptions that can introduce errors in risk assessment. In some cases, risks may be exaggerated because bioavailability of measured PAHs are overestimated, whereas at other sites, risks could be underestimated due to the role of unmeasured co-occurring hydrocarbons.

To quantify the bioavailability of total dissolved oil, a passive sampling method, referred to as biomimetic extraction (BE), has been developed (Letinski et al., 2014). This method involves equilibrating polydimethylsiloxane (PDMS) fibers with oil contaminated water samples so that aqueous concentrations of more soluble hydrocarbon components (log K*_ow_* < 6) that are expected to contribute to aquatic toxicity are negligibly depleted. The hydrocarbons that partition from the sample to the PDMS are then solvent-extracted or thermally desorbed and quantified using gas chromatography with flame ionization detection (GC-FID). This method relies on the fact that different, individual hydrocarbons exhibit similar molar response factors using FID when the instrument and acquisition method have been properly optimized.

Thus, the area of the FID response, when normalized to the molar response using an external hydrocarbon standard, provides an estimate of total dissolved oil that includes both resolvable and unresolved hydrocarbons. Further, this method can capture other nonpolar compounds (e.g., PCBs, halogenated solvents) beyond hydrocarbons that contribute to site-specific contaminant mixtures.

Concentrations of PAHs in PDMS resulting from equilibrium with contaminated sediments and soils have been shown to strongly correlate with lipid-normalized concentrations in biota as well as PAH mixture toxicity (Lu et al. 2011; Schmidt et al. 2013; Van Der Wal et al. 2004). Biomimetic extraction analytical measurements of sediments provide PDMS concentrations of PAHs and other unresolved constituents that depend on the composition and partitioning behavior of the individual mixture components in porewater. Thus, *C_pdms_*_,_ expressed on a molar basis, provides a proxy of internal mixture exposure in lipid that correlates with TUs (Mayer et al., 2014; Redman et al., 2018a). Biomimetic extraction analysis has been applied to elucidate the toxicity of complex hydrocarbon mixtures in water accommodated fractions prepared with different crude oils or petroleum products (Hedgpeth et al., 2019; Letinski et al., 2014; Redman et al., 2014; 2017) as well as produced water and refinery effluents (Redman et al., 2018b; Whale et al., 2022). Biomimetic extraction has also been shown to provide a convenient exposure metric for deriving critical PDMS concentrations that characterize oil toxicity for different test species/effect endpoints and establishing species sensitivity distributions used in risk evaluations (Redman et al., 2018a). Efforts have also been reported describing standardization and interlaboratory repeatability of BE analysis of aqueous samples (Letinski et al., 2022).

The goal of this work was to extend the BE analytical method to contaminated sediments. Our approach focused on the application of ex situ equilibrium passive sampling, which provides a simpler, quicker, and less expensive alternative to in situ deployments and avoids the need to incorporate performance reference compounds in analysis and data interpretation. This passive sampling strategy has been the focus of recent standardization efforts for determining freely dissolved concentrations of individual PAHs and PCBs in sediments (Jonker et al., 2018; 2020; Lotufo et al., 2022). Further, ex situ passive sampling has been shown to provide similar or conservative porewater concentrations when compared to in situ measurements (Apell & Gschwend, 2016; Khairy & Lohmann, 2020; Reininghaus et al., 2020; Yan et al., 2020).

The specific objectives of this study were (1) describe the BE method used for sediment analysis and explain how BE data can be translated into TUs of the unresolved, bioavailable mixture; (2) report sediment BE data collected across a range of sites with varying degrees and sources of contamination; (3) compare TUs predicted from EqP based on bulk PAH chemistry and sediment total organic carbon (TOC), or estimated, using PAH porewater concentrations or BE measurements obtained by ex situ passive sampling; (4) discuss implications of this comparative analysis for benthic toxicity assessment; (5) apply insights to construct a decision-support framework to guide contaminated sediment site assessment and remediation; and (6) recommend research priorities for framework implementation.

## Materials and methods

### Field sediments

Sediments (*n *=* *73) were collected from 17 sites (identified as A through Q) including freshwater, estuarine, and marine waterways with differing extents and sources of contamination. All sediments were analyzed for TOC and total sediment concentrations of 34 PAHs (16 parent and 18 alkyl PAHs) except one site (*n *=* *5 samples) where only 16 parent and 6 alkyl PAHs were reported. For nine sites (*n *=* *29 samples), an expanded list of 52 or 62 analytes including additional PAHs, thiophenes, and decalin homologs were also analyzed. For 19 samples collected from three of the sites, ex situ passive sampling was also performed on sediments to quantify concentrations of up to 62 PAH analytes in porewater. Ex situ passive sampling analysis using the BE method was conducted on all 73 sediments to provide comparative data.

### TOC and bulk PAH analysis

Total organic carbon was determined using EPA method 9060 (USEPA, 1988). Concentrations of PAHs in sediment samples were measured by gas chromatography-mass spectrometry-selective ion monitoring mode (GC/MS-SIM) using a modified EPA 8270E method (USEPA, 2018). These analyses are routinely available from commercial laboratories and were performed in this study by Alpha Analytical.

### PAH porewater analysis

Concentrations of PAHs in sediment porewater were determined ex situ using 36.4 µm PDMS-coated solid phase microextraction fibers (SPME) fibers (Polymicro Technologies). Prior to use, the fibers were cut into 5 cm lengths (V_pdms_ = 3 µl), washed 2 × 30 min with acetonitrile, rinsed with Milli-Q water, and dried with a Kimwipe. Polydimethylsiloxane fibers were impregnated with performance reference compounds (PRCs) to assess the fractional steady-state achieved during deployment. Stock solutions of fluoranthene-d10, chrysene-d12, benzo[b]fluoranthene-d12, and dibenz[a,h]anthracene-d14 were purchased from Cambridge Isotope Laboratories. Fibers were preloaded with the deuterated PRCs by exposure to a spiking solution with final aqueous concentrations of 30 μg/L fluoranthene, 50 μg/l chrysene, 50 μg/L benzo[b]fluoranthene, and 25 μg/L dibenz[a,h]anthracene for 14 days on a shaking table.

Sediment samples were dosed with mercuric chloride (HgCl_2_) to prevent biological activity at a concentration of 3 mg/L and homogenized on a roller bank. After this step, approximately 30 g of wet sediment subsamples were transferred into small vials (20 ml) in triplicate. Performance reference compound-loaded SPME fibers were then inserted into a Teflon septum and added to vials containing sediment (one 5 cm fiber per vial), closed, and covered with aluminum foil. Samples were allowed to equilibrate for 20 days with gentle shaking on a shaking table at 20°C. Following the equilibration period, fibers were withdrawn, cleaned with moist tissues, cut into 2 cm segments and placed in an autosampler vial with inserts filled with 250 μl of methylene chloride. Solvent blanks were included during sampler processing and transfer. The samples were stored at -20°C overnight to allow target analytes to desorb from the PDMS into the solvent. Solvent extracts were then shipped to Alpha Analytical for quantification of 62 PAHs, thiophene, and decalin analytes using GC/MS-SIM following EPA 8270D. Prior to analysis, extracts were first spiked with an internal standard containing acenaphthene-d10 and chrysene-d12 (5 mg/L). A six-point calibration was used in quantification of extract concentrations.

Extract concentrations were multiplied by the solvent volume to determine nanograms of PAH extracted and then divided by the PDMS volume extracted to determine fiber concentrations. Porewater concentrations for each analyte i were then calculated by


[1] $$C_{pw,i}=\frac{C_{\mathrm{pdms},i}}{K_{\mathrm{pdms},i\;}f_{ss,i}}$$

where *C_pdms_* is the analytically determined concentration in PDMS (µg/L_pdms_); *K_pdms_* is the PDMS-water partition coefficient (L_water_/L_pdms_); and *f_ss_* is the fraction of steady-state achieved. *K_pdms_* was estimated from log *K_ow_* using the regression reported by Reininghaus et al. (2020) that included a range of parent and alkyl PAHs (log *K*_pdms_ =0.831 × log *K_OW_* + 0.13, r^2^ = 0.89). The *f_ss_* were determined by fitting the observed PRC data to the mass transfer model by Thomas and Reible (2015) to yield a relationship in which *f_ss_* for PAH analytes can be estimated from log *K_ow_* as described in Burgess (2012).

### Translation of predicted or measured porewater concentrations into toxic units

Toxic units were calculated from bulk PAH analysis and sediment TOC using EqP theory:


[2] $$\mathrm{TU}\;{\mathrm{PAH}}_{n}\;\mathrm{EqP}=\sum_{i=1}^{n}[C_{\mathrm{soc},i}/K_{\mathrm{oc},i}]/{\mathrm{FCV}}_{i\;}$$

where $C_{\mathrm{soc},i}$ = organic carbon normalized sediment concentration for PAH_i_ (mmol/kg oc); $K_{\mathrm{oc},i}$ = sediment organic to water partition coefficient for PAH_i_ (L/kg oc); ${\mathrm{FCV}}_{i}\;$= final chronic value in porewater for benthic life protection for PAH_i_ (mmol/L); where the subscripts *i* and *n* denote the individual analytes targeted and the corresponding number of analytes included in the TU sum.

Toxic units were also computed using measured porewater concentrations derived from ex situ passive sampling measurements:


[3] $$\mathrm{TU}\;{\mathrm{PAH}}_{n}\;\mathrm{PS}=\sum_{i=1}^{n}[C_{\mathrm{pw},i}]/{\mathrm{FCV}}_{i}\;$$

where $C_{\mathrm{pw}}$ is the measured porewater concentration for analyte *i* obtained using *Equation 1* from passive sampling data and *n* is the number of analytes included in the TU sum.

Final chronic values were originally derived from an early calibration of the TLM (Burgess et al., 2013). The FCVs used in our study have been recomputed using the updated TLM reported by McGrath et al. (2018) as summarized in online supplementary material Table S1 for the 62 target analytes. *K*_oc_ values for these analytes were estimated using the regression with log *K*_ow_ reported by Burgess et al. (2013) and are listed in online supplementary material Table S1.

### Ex situ BE analysis

Spools of 30 µm PDMS coated fibers (Polymicro Technologies) were cut into 5 or 6 cm lengths and precleaned by either thermal desorption at 320°C or soaking consecutively with three solvents, i.e., dichloromethane (2×), acetone (2×) and methanol (2×) for 30 minutes each. After the methanol solvent wash, the fibers were rinsed with Milli-Q water. The rinsed PDMS fibers are then blotted dry with lint-free tissues.

Replicates for BE analysis consisted of adding 15–25 g wet homogenized sediment in 20 ml glass amber vials with Teflon lined caps. The contents were poisoned with 0.25 ml of 1% HgCl_2_ or 100 ppm of sodium azide (NaN_3_) to prevent potential biodegradation during fiber equilibration but not acidifed. Typically, two or three replicate vials were prepared for each sediment sample. The vials were rolled overnight at 20 rpm on an IBI Scientific Low-Profile Roller. The next day, two fibers (PDMS volume = 0.8 µl per fiber) were added to each vial with rolling continued for 21–28 days at approximately 20°C. Preliminary studies have shown this duration is sufficient to achieve equilibrium across a range of field sediments using the quantitation method used (see below) consistent with other studies where mixing is applied (Lotufo et al., 2022). Although faster kinetics can be achieved with 10 µm fibers to shorten the equilibration period, use of thinner fibers reduces sensitivity, can result in losses of the more volatile PAH components, and has been found to decrease precision of replicate measurements. The current protocol adopted for BE analysis provides a ratio of sediment organic carbon to PDMS that is intended to provide an extraction that is negligibly depletive (Mayer et al., 2014), consistent with the typical ratios of biota to sediment in toxicity tests.

Following the equilibration period, fibers were removed, rinsed with distilled water, and blotted dry. Fibers were extracted by either thermal desorption using a CTC autosampler (GC sampler 120 from Agilent Technologies), or by solvent extraction with 0.25 ml acetone-methylene chloride and analyzed using large volume injection GC-FID using an injection volume of 20 μl. A set of five individual hydrocarbons (toluene, o-xylene, 2-methyl naphthalene, 2,3-dimethyl naphthalene, and 9-methyl anthracene) were prepared in methylene chloride at concentrations ranging from 0.01 to 10 μg/ml. *C_pdms_* was estimated by translating the area under the FID curve into molar units using the molar response of 2,3-dimethylnaphthalene as determined by injection of external standards. The estimated amount of nmols were then normalized by the PDMS fiber volume so that results are expressed in units of nmoles/µl _PMDS_ = µmoles/ml _PMDS_ = mM. The practical quantification limit of this method is 0.2 mM based on the response of the lowest analyzed BE standard, the PDMS fiber volume, and the solvent extraction volume used to desorb the fiber.

### Fluorometry analysis

Vials containing extracts from ex situ passive sampling used for sediment BE analysis were subsampled and used for fluorescence measurements in low volume cuvettes (700 ul) for a subset (*n* = 11) of sediment samples collected from six sites investigated in this study. Initially, an excitation emission matrix (EEM) scan for a solvent blank was performed using an automated fluorescence spectrophotometer (HORIBA Aqualog). The same approach was used to determine the optimal excitation-emission couples for each sample extract. The excitation and emission scan ranges overlap in certain wavelength regions, which manifest in signals from the scattered light. Therefore, the EEM collected from the solvent blank was subtracted from the sample EEM to remove the Raman scatter lines. Following this step, a Rayleigh-masking algorithm was applied to remove the first and second order Rayleigh lines. The maximum intensity for each excitation-emission couple was recorded and compared with the corresponding BE concentration determined for each investigated sediment.

### Translation of BE measurements into toxic units

BE measurements can be directly converted into estimated toxic units:


[4] $$\text{BE}\;\text{TU}={\;C}_{\text{pdms}}/{\text{FCV}}_{\text{pdms}}$$

where C_pdms_ = concentration in passive sampler equilibrated with sediment which serves as the BE measurement (µmol/ml_pdms_); and FCV_pdms_ = final chronic value for total bioavailable hydrocarbons (µmol/ml_pdms_)

Critical fiber concentrations corresponding to acute effects (i.e., median lethal concentrations/half-maximal effective concentrations [LC/EC_50_s]) for different test species have been determined from observed oil toxicity data and companion BE measurements (Redman et al., 2018a). These data were used to calculate the fifth percentile of PDMS concentrations corresponding to the final acute value (FAV_pdms_) = 14.2 μmol/ml_pdms_. A geometric mean acute to chronic ratio (ACR) of 2.9 was also calculated from this study. The FCV_pdms_ of ∼ 5 μmol/ml_pdms_ was derived by dividing the FAV_pdms_ by the ACR (i.e., 14.2/2.9)_._ Thus, TU BEs were conveniently estimated by dividing the measured BE by this value.

## Results

### TOC and bulk PAH analysis

**Figure 1.**
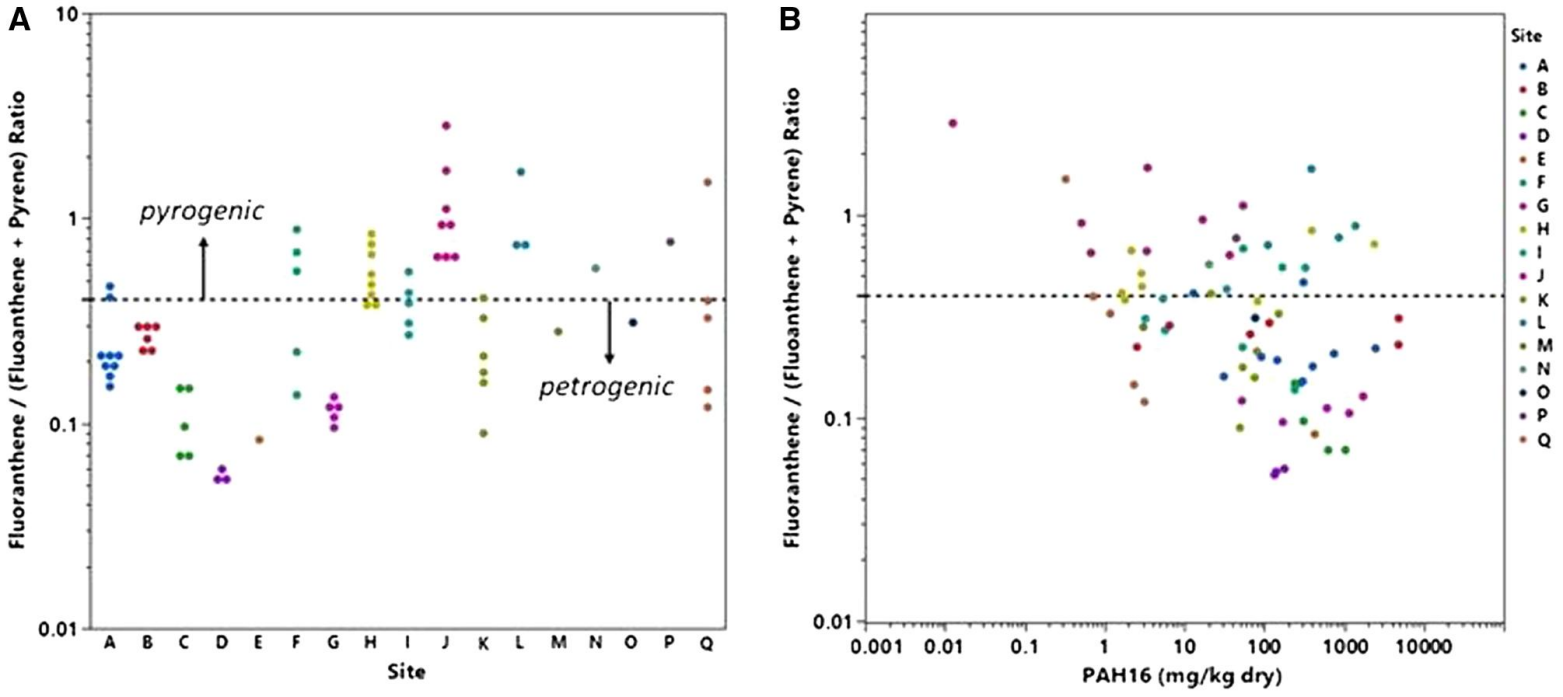
A: Diagnostic source ratios for samples collected at different study sites. B: Diagnostic ratios plotted as a function of total polyaromatic hydrocarbon (PAH)16 bulk sediment concentrations.

Total organic carbons and bulk sediment concentrations of individual PAHs and related hydrocarbons are reported in online supplementary material Table S2. Sediment TOC (%) averaged 4.38 and ranged from 0.37 to 16.90 across the 73 test sediments. The ratio of fluoranthene/(fluoranthene + pyrene) sediment concentrations has been used as diagnostic tool for identifying petrogenic and pyrogenic sources (Cao et al., 2020; Kieta et al., 2022) and is shown plotted in Figure 1A for the 17 sites included in this study. Results suggest samples reflect contamination from various PAH sources. This ratio is plotted as a function of the PAH16 concentration for the various samples to highlight the over six-order magnitude variation in PAH contamination that is represented by this dataset, i.e., 0.01 to 4,821 mg/kg_dry_. A two-dimensional plot with a second diagnostic ratio, benzo(a)anthracene/[benzo(a)anthracene + chrysene, is provided in online supplementary material Figure S1 to further illustrate the different PAH sources associated with individual samples within and between sites. The analysis shows that the source and composition of the PAH in these sediment samples varied substantially, providing a robust dataset for evaluating the proposed decision-making framework presented below.

### TUs from EqP predictions

**Figure 2.**
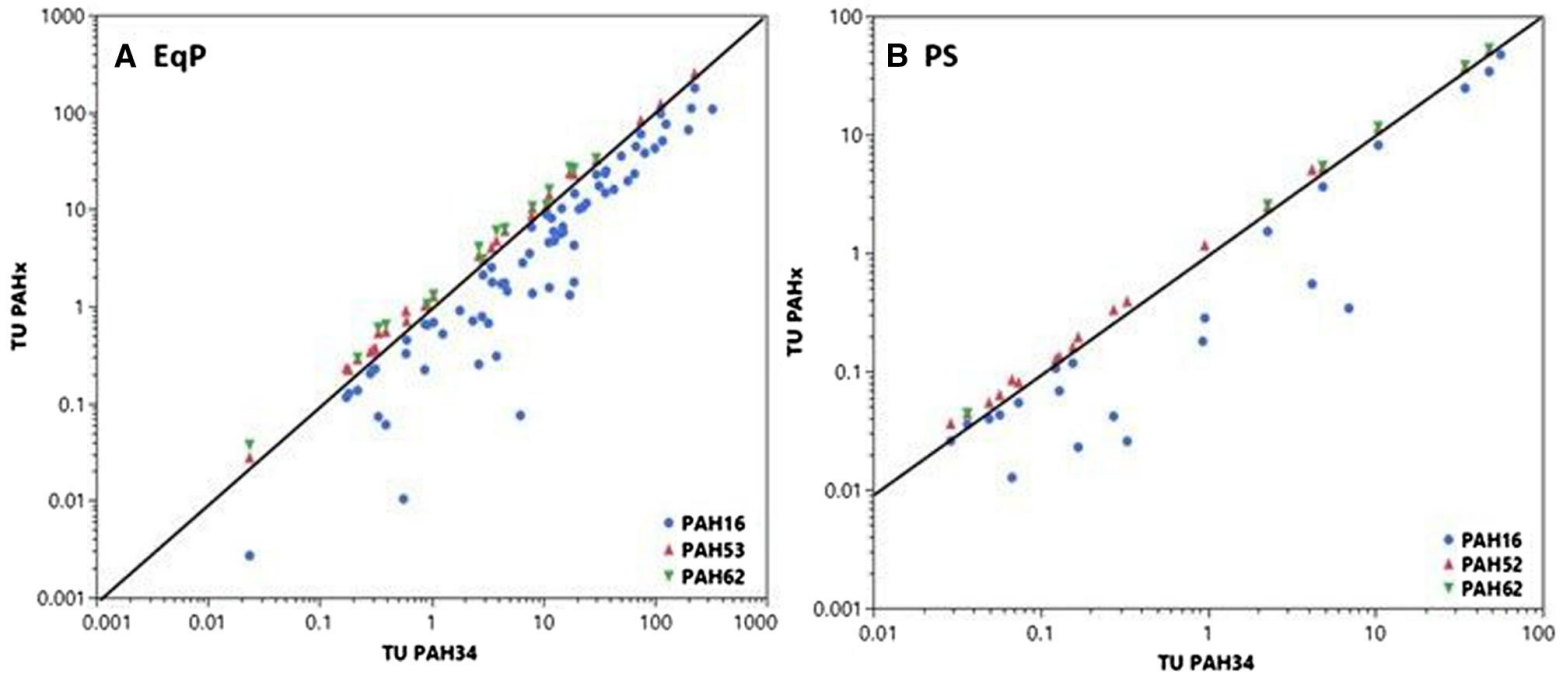
Comparison of toxic units computed using polyaromatic hydrocarbon (PAH)16, 52, or 62 with PAH34 based on (A) predicted porewater concentrations based on equilibrium partitioning calculations or (B) measured porewater concentrations based on passive sampling measurements.

Toxic units (online supplementary material Table S3) were computed from sediment organic carbon-normalized sediment concentrations (online supplementary material Table S2) and FCVs (online supplementary material Table S1) using *Equation 2* without consideration of solubility cut-offs (USEPA, 2012). Toxic units were computed based on the sum of 16 priority pollutant PAHs, as well as an expanded list of 34, 52, and 62 target analytes. Increasingly, PAH34 is often the key metric used in evaluating PAH sediment contamination because it includes both parent and alkyl PAHs that capture both pyrogenic and petrogenic sources. Toxic unit PAH34 EqP could be calculated for 68 samples and ranged from 0.02 to 328. Thus, this dataset includes samples expected to be both nontoxic and toxic to benthos. Toxic units calculated using 16, 52, and 62 PAHs were also calculated and are shown cross-plotted with TU PAH34 in Figure 2A. Results indicate that PAH16 denoted by the blue symbols in Figure 2A can underestimate TUs derived using PAH34 by over an order of magnitude for some sediment samples. In contrast, TUs derived using PAH52 or PAH62 denoted by the red and green symbols, respectively, are well correlated with PAH34. The TU PAH52/PAH34 ratio averaged 1.22 (range 1.03–1.59, *n *=* *29) whereas the TU PAH62/PAH34 ratio averaged 1.39 (range 1.03–1.80, *n *=* *16). Thus, based on these results, PAH34 appears to provide a robust estimate of PAH sediment contamination, as inclusion of up to 28 additional analytes only marginally increase estimated TU.

### Porewater measurements from ex situ passive sampling

Porewater measurements were obtained from passive sampling data collected for a subset of 19 sediment samples (online supplementary material Table S4). For comparison, predicted porewater concentrations for the same sediments derived via EqP are also provided in online supplementary material Table S4. Resulting TUs computed from measured porewater concentrations using *equation 3* are summarized in online supplementary material Table S5 for different PAH sum metrics. Additional bulk sediment and ex situ passive sampling porewater concentrations of PAHs have been reported in two other studies. In the study by Conder et al. (2021), TOC and PAH34 bulk sediment analyses and measured porewater concentrations were reported for three sediment samples (sample C=low petroleum contamination, G2 = high petroleum contamination, and V1 = creosote contamination). In the study by Reininghaus et al. (2020), TOC and PAH25 bulk sediment analyses and corresponding measured porewater concentrations were provided for six sediment samples collected from the Baltic Sea. Equilibrium partitioning predicted and measured porewater concentrations derived using ex situ passive sampling from these studies are summarized in online supplementary material Table S6 with corresponding TUs provided in online supplementary material Table S7.

Measured porewater concentrations obtained by passive sampling are shown cross-plotted with EqP predictions for different target analyte classes in online supplementary material Figure S2A–E. In these figures, data from samples collected from different sites are denoted with different colored symbols. The sites from this study correspond to H, I, and J whereas data included from the two additional literature studies are denoted by R and S in the figure legend. Results indicate that predicted and measured porewater concentrations for two-ring PAHs are in reasonable agreement, as datapoints fall above and below the 1:1 line (online supplementary material Figure S2A). However, there is a trend of increasing positive bias in higher EqP predictions with increasing PAH ring number (online supplementary material Figure S2B–D). This bias is most apparent for the five-ring PAHs, as predicted concentrations systematically overstate measured concentrations by an order of magnitude on average. The limited bioavailability of higher PAHs may reflect the preferential partitioning to black carbon as previously reported (Lohmann et al., 2005). Less data are available for benzothiophene compounds, but predictions tend to overstate measured porewater concentrations by generally less than an order of magnitude (online supplementary material Figure S2E).

### TUs from measured porewater concentrations

Toxic units were calculated using measured porewater concentrations based on the sum of 16, 34, 52, or 62 target analytes. Toxic unit PAH34 PS could be calculated for 19 samples and averaged 5.6 with a range of 0.03 to 47.9. Thus, this subset includes samples expected to be both nontoxic and toxic to benthos. Toxic units calculated using 16, 52, and 62 PAHs are cross-plotted with TU PAH34 in Figure 2B. Results indicate that PAH16 can underestimate TUs derived using PAH34 by over an order of magnitude for some sediment samples. In contrast, TUs derived using PAH52 or PAH62 are well correlated with PAH34. The TU PAH52/PAH34 ratio averaged 1.13 (range 1.02–1.26, *n *=* *19) whereas the TU PAH62/PAH34 ratio averaged 1.11 (range 1.02–1.24, *n *=* *13). Thus, consistent with findings discussed previously using EqP calculations, PAH34 appears to provide a robust estimate of PAH sediment contamination.

### Comparison of TUs

**Figure 3.**
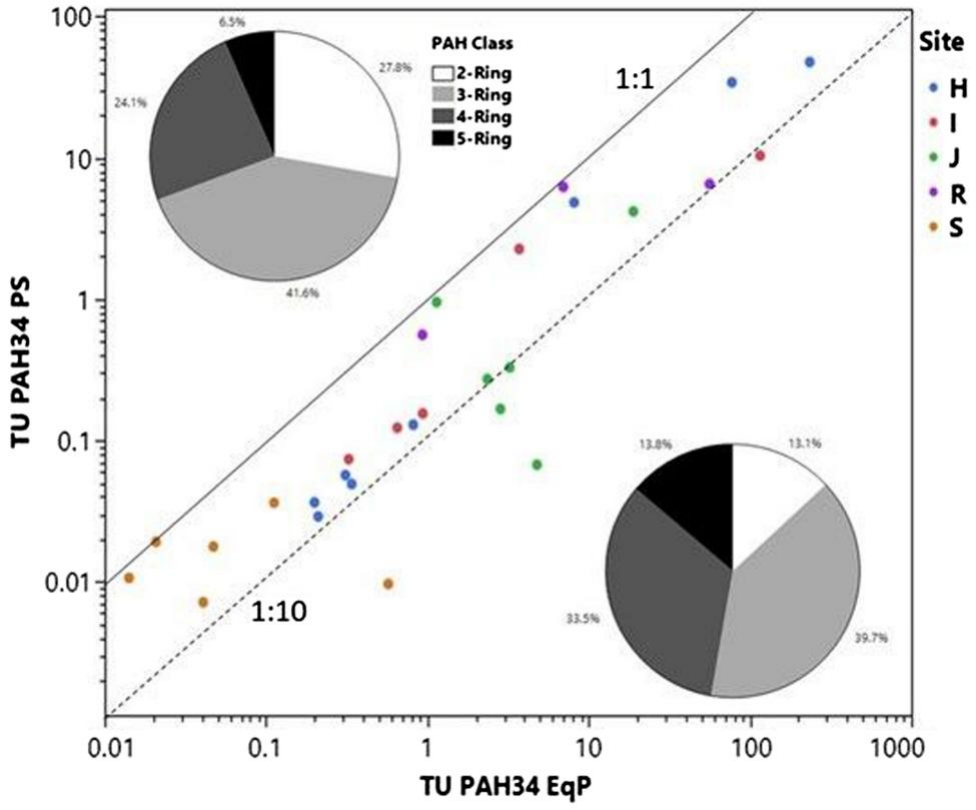
Comparison of toxic units computed from passive sampling (PS) measurements of sediment porewater with values derived using equilibrium partitioning (EqP). Toxic units based on the sum of 34 polyaromatic hydrocarbons (PAHs) except for site S where 25 PAHs were used.

Toxic units derived using PAH34 based on EqP predictions (online supplementary material Table S3) or PS data (online supplementary material Table S5) for the subset of samples where both metrics are available along with comparative data for the two additional studies discussed above (online supplementary material Table S7) are shown plotted in Figure 3. For the Reininghaus et al. (2020) study (denoted as site S), only 25 PAHs were reported, so this sum metric is included in this plot. Toxic units derived via PS using measured porewater concentrations appear systematically lower than TU EqP, but 25 out of 28 sediment samples (i.e., 89%) were found to fall within an order of magnitude as indicated by plotting above the 1:10 dashed line. For the 22 sediments where both TU PAH34 EqP and PS metrics could be determined, the mean percentage of contribution of different PAH ring classes to TUs is displayed as pie charts in Figure 3. Comparing these pie charts reveals that EqP-based TU predictions reflect a greater contribution of four- and five-ring PAHs (42%) than the passive sampling-based TU metric (31%). This is consistent with the reduced bioavailability of the higher molecular weight PAHs discussed earlier. In 21 out of 22 samples, two- to three-ring PAHs contribute a higher percentage of TUs associated with passive sampling measurements than EqP predictions (online supplementary material Figure S3). The one exception is a creosote sample included in the study of Conder et al. (2021), where two- + three-ring PAHs contributed about 80% of the TUPAH34 EqP but only approximately 50% for TU PAH34 PS. This discrepancy could be attributable to losses of the most volatile two-ring PAHs from 13 μM polyethylene passive samplers prior to analysis, which were present at high concentrations in bulk sediment (online supplementary material Table S6). Such losses would bias measured porewater concentrations low relative to EqP predictions.

Burgess et al. (2021) evaluated TU PAH16 using both EqP predictions and porewater concentrations derived from in situ passive sampling measurements for six sediments at a gas manufacturing superfund site. The mean ratio of TUPAH16 EqP/PS was reported to be 15.1 (range 1.7–54.4; *n* = 6). These results compare favorably with this study (online supplementary material Table S5) and earlier literature (online supplementary material Table S7), in which an average ratio of 13.5 (range 1.7–115.5; *n* = 19) and 20.5 (range 1.4–105.5; *n* = 9), respectively, were obtained. Thus, based on these collective studies, the TU PAH16 EqP metric overstated bioavailability, on average, by more than an order of magnitude and exhibited a two order of magnitude range across different sediments.

### Ex situ BE measurements

Representative GC-FID chromatograms of the BE standard and two sediment samples are illustrated in online supplementary material Figure S4. Molar response determined by FID was linear over the calibration range (online supplementary material Figure S5) and the slope of this relationship was used to determine the molar response factor for translating the FID sample response to molar concentrations extracted from the fiber. The BE method is intended to quantify aromatic and aliphatic hydrocarbons in approximately the 3 to 7.5 log K_*ow*_ range. This is the range of hydrocarbons that is most relevant for aquatic toxicity (Parkerton et al., 2021). This includes aliphatic hydrocarbons up to approximately 15 carbons and aromatic hydrocarbons up to approximately 20 carbons. Compounds containing both aliphatic and aromatic functional groups will have carbon numbers captured by BE analysis that fall within this range. Higher log K_*ow*_ compounds do not significantly contribute to the GC-FID signal of the BE measurements due to both solubility constraints and kinetic limitations of the passive sampler, which precludes equilibrium over the time frame deployed (e.g., 21–28 days). The molar response of individual hydrocarbons using FID normalized to 2,3-dimethyl naphthalene increases as a function of GC retention time over the relevant carbon numbers discussed above by a factor of approximately two. This provides justification for using 2,3-dimethyl naphthalene, which falls in the middle of this range, to translate FID response into molar units.

Other nonpolar chemicals in the relevant log K_*ow*_ range such as halogenated or oxygenated compounds (e.g., chlorinated benzenes, PCBs, polychlorinated dibenzodioxins [PCDDs], polybrominated diphenylethers [PBDEs], phthalates) and hydrocarbon degradation products (e.g., oxy-PAHs, alcohols) may also in contribute to the BE signal if present in the sediment. However, in general, the significance of these other non-hydrocarbon classes is expected to be limited, given generally lower sediment concentrations compared with hydrocarbons coupled with the lower molar response factors associated with these classes using FID (Tong & Karasek, 1984). Compounds such as organic acids, diols, and ethoxylated alcohols are too polar to be efficiently extracted using PDMS and are not expected to be captured in BE measurements. Thus, BE analyses are intended to provide an estimate of the total bioavailable hydrocarbons present in a contaminated sediment.

**Figure 4.**
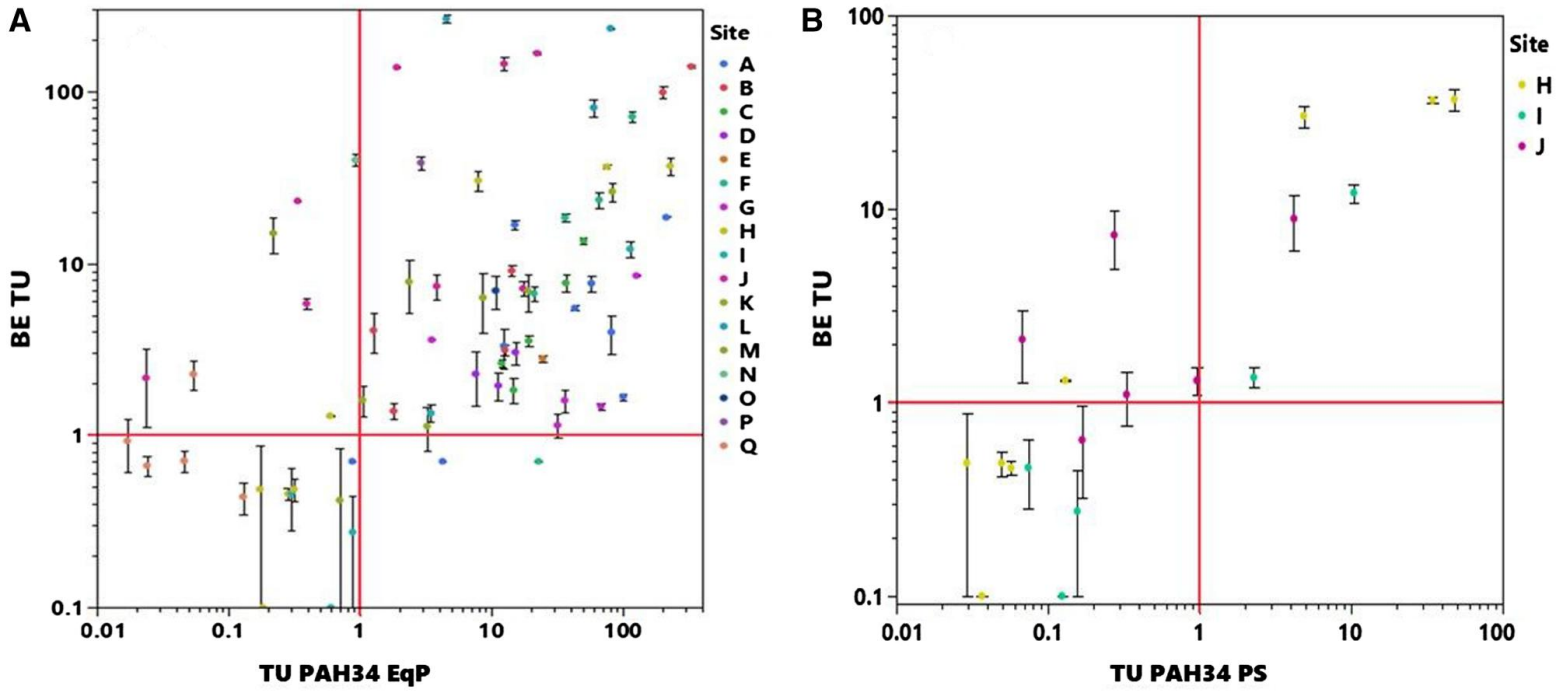
Correlation of toxic units derived from ex situ biomimetic extraction sediment analysis with predictions from (A) equilibrium partitioning and (B) passive sampling based on polyaromatic hydrocarbon (PAH)34.

Sediment BE values for the 73 test sediments averaged 110.9 and ranged from 0.5 to 1,164 μmol/ml_pdms_ (online supplementary material Table S8). To provide an indication of method precision, the average coefficient of variation for this method based on the standard error of replicate BE analyses for a given sediment sample was 6.3% (online supplementary material Table S8). Biomimetic extraction TUs (online supplementary material Table S8) were derived by applying *equation 4* and are shown plotted as a function of TU PAH34 EqP in Figure 4A and TU PAH16 EqP in online supplementary material Figure S6. A positive correlation between these two different TU metrics is apparent. A 88% concordance between BE TU and TU PAH34 EqP was obtained in identifying sediments with TUs either less than or equal to 1 (Table 1). In 8% of the samples, BE TU were > 1 and TU PAH 34 EqP < 1, whereas in 3% of the samples, BE TU < 1 and TU PAH34 EqP >1. Approximately one-third of the sediment tested exhibited BE TUs that exceeded TU PAH34 EqP. For several sites (J, L, M, N, P, and Q), BE TUs were more than an order of magnitude higher than EqP calculations (online supplementary material Figure S7). These sites tend to have more pronounced petrogenic source signatures (c.f. Figure 1). Further analysis was performed to investigate if alkyl-PAHs or PAH ring composition derived from EqP calculations correlated with BE measurements. However, no clear trends were observed.

**Table 1.** Contingency table summarizing biomimetic extraction (BE) and equilibrium partitioning polyaromatic hydrocarbon 34 toxic unit (EqP PAH34 TU) metrics for 68 site sediments. Values represent percentage of samples falling in each quadrant (A–D) shown in Figure 5.

	EqP TU PAH34 < 1	EqP TU PAH34 > 1
**BE TU < 1**	A = 18%	B = 3%
**BE TU > 1**	D = 8%	C = 70%

It is also instructive to consider the empirical relationship between BE TUs and TU PAH34 determined using passive sampling measurements. Although fewer data were available for this metric across only three sites, TU PAH34 PS was shown to be approximately equal to or greater than BE TU (Figure 4B). This is expected as the BE TU metric captures the bioavailable contribution of all 34 PAHs. Therefore, these results provide an independent check on the intended performance of the BE method and corresponding TU derivation approach.

### Fluorometry measurements

Additional analytical approaches to GC-FID that can be coupled with ex situ passive sampling may offer even simpler analytical tools for rapid sediment screening. Two potential approaches include biosensor and fluorometric methods. The former approach has shown promise for porewater analysis (Conder et al., 2021), whereas the latter has been successfully applied for screening bulk sediment PAH wet concentrations (Burgess et al., 2021). A preliminary evaluation of fluorometric analysis of BE sediment extracts was performed in this study using 11 sediments collected from six sites. The optimal excitation wavelength for fluorometric response fell within a narrow interval from 332 to 335 nm with the corresponding emission wavelength in the range of 365 to 381 nm (online supplementary material Table S9). The observed fluorometric intensity of extracts was well correlated to the corresponding BE concentrations determined for these sediments (online supplementary material Figure S8). These results suggest that coupling ex situ passive sampling with fluorometric analysis could serve as a powerful analytical screening tool for more rapid and cost-effective evaluation of PAH bioavailability in site characterization of sediments.

## Discussion

### Implications of toxic unit analyses

Studies that incorporate empirical passive sampling data into models for toxicity prediction have been identified as a research priority (Burgess et al., 2023). This study addresses this need by evaluating PAH contaminant mixtures in sediments using three different TU metrics based on bulk chemistry and ex situ passive sampling data collected across a wide range of contaminated sites. The proposed TU metrics serve as chemical-based lines of evidence for assessing potential toxicity to benthic life. This analysis indicates that PAH34 provides a good proxy of estimated TUs derived using a more extended target list of up to 62 analytes. Further, EqP-based TU PAH34 can overstate the toxic potential of PAH contaminated sediments by up to an order of magnitude due to enhanced partitioning in field sediments that reduces PAH bioavailability and resulting porewater concentrations. In contrast, TUs derived from BE measurements, which also are intended to integrate sediment bioavailability considerations, were greater than TU PAH34 EqP predictions for about one-third of the sediment samples investigated in this study. This apparent inconsistency may be due to several reasons. First, other unquantified nonionic sediment contaminants, such as compounds present in the UCM that partition from sediment to porewater and can absorb to the fiber, may contribute to the GC-FID signal and resulting TU estimates. The potential hazard of UCM components to both aquatic and sediment-dwelling organisms has been recognized (Petersen et al., 2017; Scarlett et al., 2007) but not typically quantified in contaminated sediment risk evaluations (Ramadass et al., 2021).

Second, the higher magnitude of BE TUs when compared with TU PAH34 EqP predictions could reflect fiber fouling (e.g. oiling), particularly for the samples with the highest BE measurements. To further explore this hypothesis, sediments from site L, which exhibited among the highest measured BE and is known to be contaminated with nonaqueous phase liquid (NAPL), were evaluated using 30 μm PDMS fibers either directly placed in sediment (consistent with BE protocol described earlier in the method section) or alternatively deployed within a stainless steel wire mesh. This later modification was intended to prevent direct contact of fiber with sediment and thereby mitigate potential fouling. Although only two sediment samples were investigated, a 59% to 86% reduction in 28-day BE results were observed when fibers were deployed in the wire mesh (Table 2). These results suggest the high BE values reported at this highly contaminated site may indeed be confounded by fiber fouling. However, an examination of BE fiber chromatograms with the mesh indicates that although the magnitude of the response is reduced, the shape of the chromatogram is largely unchanged. If the mesh was preventing fouling, one would expect that the heavier components would be preferentially reduced. To confirm the fouling hypothesis, additional ex situ passive sampling targeting individual PAHs could be performed. If subsequent passive sampling data indicate that estimated porewater concentrations exceed aqueous solubility limits, these results would be diagnostic of NAPL interference.

**Table 2.** Ex situ biomimetic extraction (BE) measurements performed for two sediment samples collected from site L in which fibers were deployed either directly into the sediment or incorporated into a wire mesh holder.

Sample	Fiber deployment	Equilibration time (days)	BE (μmol/ml_pdms_)	Fiber deployment	Equilibration time (days)	BE (μmol/ml_pdms_)
1	No mesh	14	355.5 ± 61.9	Wire mesh	14	79.6.5 ± 8.1
			*n* = 3			*n* = 4
		28	461.8 ± 61.9		28	169.3 ± 35.9
			*n* = 3			*n* = 4
		42	Not tested		42	216.5 ± 36.7
						*n* = 4
2	No mesh	14	413.5 ± 17.4	Wire mesh	14	41.9 ± 14.8
			*n* = 3			*n* = 5
		28	418 ± 22.0		28	56.2 ± 5.4
			*n* = 2			*n* = 3
		42	Not tested		42	70.4 ± 8.6
						*n* = 3

Another potential reason for higher TUs derived using BE rather than EqP calculations in less contaminated sites is the potential contribution of naturally occurring organic matter. Although the analytical detection limit of the BE method is ca. 0.2 μmol/ml_pdms_ which would correspond to a lower bound estimate of 0.04 TUs, relatively uncontaminated sediments may have a background signal that contributes to the observed FID response. Thus, background interference may be an analysis artifact that inflates estimated BE TUs. This concern is most apparent for site Q, which exhibited low TU EqP based on the measured PAH25 (online supplementary material Table S5) but much higher BE values (online supplementary material Table S8), which resulted in ratios above 10 in four out of the five samples (online supplementary material Figure S7). Further, sample 67 from this site exhibited the highest EqP TUs (online supplementary material Table S5) and was found to be the only sample with a BE TU/TU PAH25 EqP ratio < 10. This trend is consistent with the potential confounding influence of a background BE concentration in sediments with low level contamination.

### Proposed management and decision-making framework

**Figure 5.**
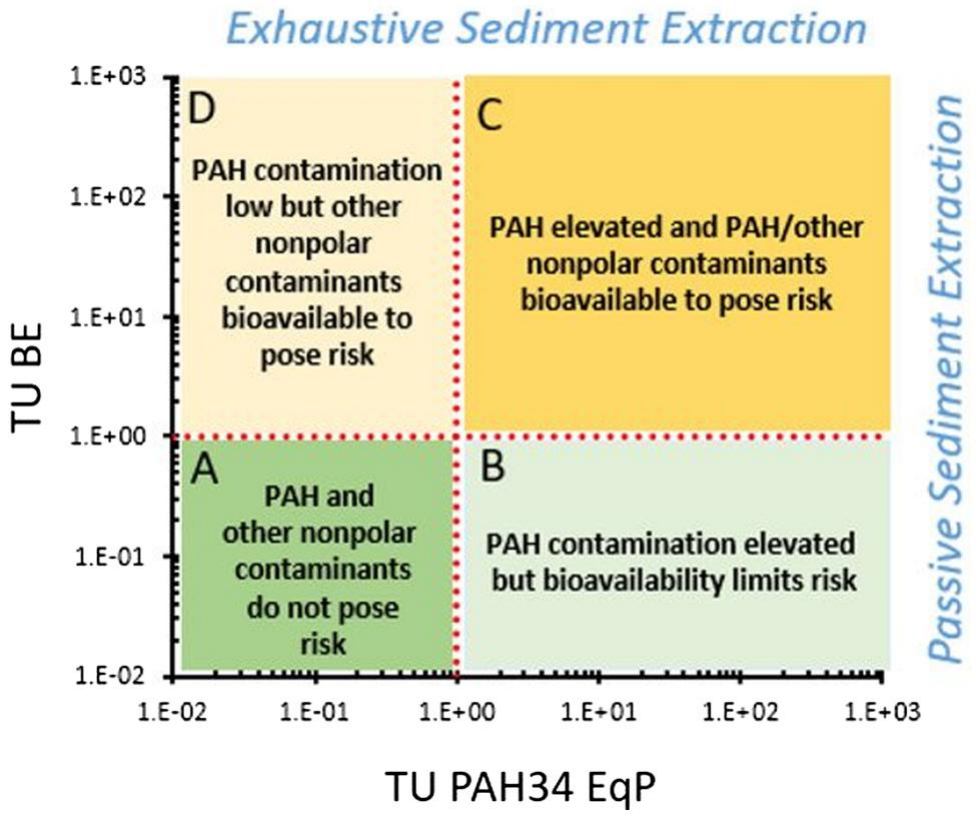
Proposed conceptual framework that integrates conventional chemical analysis of polyaromatic hydrocarbon (PAH)34 and ex situ passive sampling of sediments using the biomimetic extraction method.

Based on this analysis, we propose using TU PAH34 EqP and BE TU metrics for evaluating PAH-contaminated sediments. The EqP-based TU metric relies on conventional TOC and bulk sediment analysis, can be readily performed, and serves as a logical first screening tier in contaminated site assessment (Burgess et al., 2021; McGrath et al., 2019). If TU PAH34 EqP is > 10, it is likely that the sediment is sufficiently contaminated to pose a toxicity concern and further ex situ passive sampling to provide a more refined chemical line of evidence may not be warranted given the added effort and expense. For sediments in which TU PAH34 EqP is < 10, a second tier of sediment analysis is proposed in which ex situ passive sampling is performed to allow determination of BE TUs. This method is recommended because it simpler and less costly than ex situ measurements of individual PAHs and provides additional conservatism in capturing other contaminant mixture components that may be present beyond the limited set of 34 targeted PAHs. The results of this twofold approach for deriving TU metrics for a given sediment can then be cross plotted to guide decision-making, as illustrated in Figure 5. For sediments that fall in quadrant A or B, sediments are deemed to exhibit PAH contamination that is either too low or not bioavailable to pose benthic toxicity. For sediments that fall in quadrant C, PAHs are judged to be sufficiently high and bioavailable to pose a toxicity concern. For sediment samples in quadrant D, other contaminants beyond PAH34 may be sufficiently bioavailable to pose potential toxicity to benthic life. For sediments that fall in either quadrants C or D, further higher tier sediment toxicity testing may be considered, as suggested by Burgess et al. (2023). For sediments confirmed to be toxic to benthic life, use of in situ amendments, such as activated carbon or biochar (Yan et al., 2020; Yang et al., 2021) to reduce contaminant bioavailability, warrant consideration as a potential viable remedial option.

**Figure 6.**
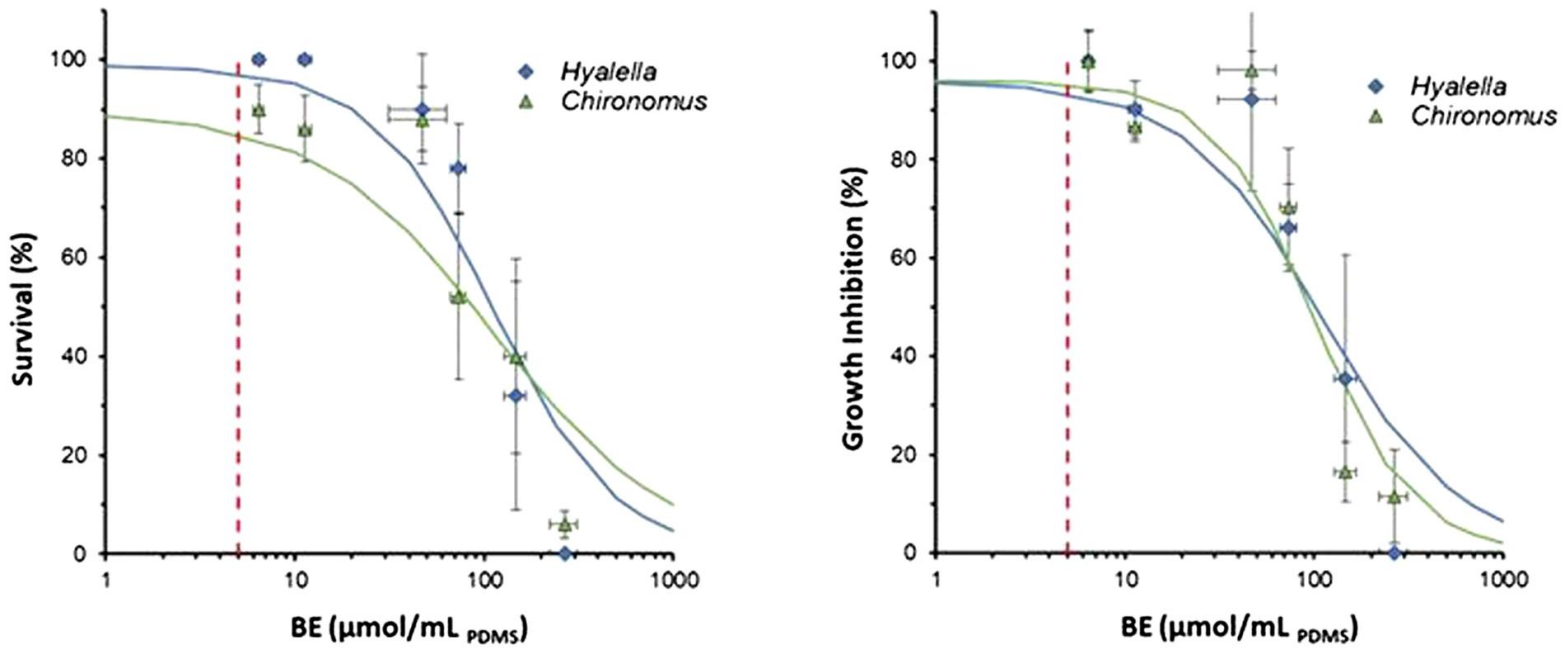
Observed biomimetic extraction concentration-response relationships for No. 2 fuel oil sediment toxicity for two freshwater test species. Panel A shows response for survival and panel B shows growth. The red dashed vertical line denotes the final chronic value.

It is important to highlight that the proposed decision framework adopts the FCV (i.e., the fifth percentile of the species sensitivity distribution) as the basis for TU calculations. If concurrent site-specific sediment toxicity data have been collected, it is often of interest to assess the concordance between chemical and toxicity testing lines of evidence to elucidate causality. In such cases, it may be useful to revise TU metrics to correspond to the critical target lipid body burden of the test species/endpoint investigated (McGrath et al., 2019). To provide a consistent basis to derive BE TUs, a corresponding BE toxicity threshold would be required (i.e., replace FCV_pdms_ with *C*_pmds, critical_ in *equation 4*). To illustrate how a BE threshold could be derived from toxicity test data, the study by Mroz (2011) was used. In this work, No. 2 fuel oil was spiked to formulated sediment at five nominal concentrations ranging from 50 to 800 ppm. Parallel BE measurements on these treatments were performed using the method previously described. The observed effects on survival and growth of two freshwater benthic test species are shown plotted as a function of sediment BE measurements in Figure 6. These data were further evaluated using the dose-response curve (drc) package in R statistical software (R V3.4.3) (Ritz et al., 2015) to derive effect endpoints (Table 3). The resulting toxicity thresholds are an order of magnitude higher than the estimated FCV_pdms_ used in earlier BE TU calculations and highlight the conservative nature of the proposed framework presented above. This analysis also illustrates how, given relevant sediment toxicity and BE data, the BE TU metric can be adjusted to reflect observed site-specific sediment toxicity test responses for a given benthic species and effect endpoint. Further, performing BE analysis in conjunction with sediment toxicity testing is expected to increase confidence in applying this method for improved exposure characterization of sediment contaminant mixtures (Grundy et al., 2023).

**Table 3.** No. 2 fuel oil toxicity results expressed in terms of measured biomimetic extraction concentrations for two benthic test species.

Test organism	Test duration (days)	Survival LC50 (μmol/ml_pdms_)	Growth EC10 (μmol/ml_pdms_)
*Hyalella azteca*	14	112.1	49.2
		(81.3–142.8)	(12.4–85.1)
*Chironomus dilutus*	10	111.3	55.7
		(67.9–154.6)	(38.5–72.8)

*Note:* LC = lethal concentration; EC = effective concentration

### Research opportunities

To advance the proposed framework presented in Figure 5, further efforts are warranted to standardize the sediment BE method, analogous to aqueous BE analyses (Letinski et al., 2022). Although the BE method outlined in this study does not include performance reference compounds to correct for equilibrium, the inclusion of a reference sediment or laboratory spiked sample of a hydrocarbon mixture to assess BE method performance is suggested. This was not done in the current work and should be considered in future standardization efforts. Future work should also include interlaboratory comparisons that focus on the accuracy and precision of sediment BE measurements and address protocol modifications to identify and potentially mitigate fiber fouling in highly contaminated sediments. Initial tests included in this study revealed that fibers enclosed in a wire mesh yielded lower sediment BE concentrations than unprotected fibers when exposed to a highly contaminated sediment. Although it appears this modification may have retarded fiber fouling, it is uncertain whether such a strategy fully mitigates the potential confounding influence of oiling. As previously mentioned, additional ex situ passive sampling of individual PAHs could be performed to assess whether concentrations are above or below water solubility limits when applying this modification. Testing less-contaminated sediments without oil using a mesh would also enable the influence of this proposed modification on BE measurements to be determined in the absence of fouling.

The role of NAPL on both BE measurements and toxicity to sediment-dwelling organisms clearly warrants further study. Recent work has proposed a sediment toxicity testing strategy for identifying effects associated with physical effects from NAPL using different benthic organisms with different susceptibilities to oiling (Jonker & Diepens, 2024) and such test designs could benefit from integration of BE analyses. Further evaluation is also needed to establish minimum detection limits for BE analyses of less contaminated sediments and determine whether potential corrections are required to account for natural background contributions to the BE signal. Additional work is also required to understand the advantages and limitations of using fluorometry as a simpler, cheaper alternative to GC-FID analysis for BE quantification. Whereas the BE measurement is mechanistically similar to processes that control the bioaccumulation and toxicity of nonpolar organic chemicals (e.g., lipid water partitioning), fluorometric detection is based on the excitation and emission of aromatic constituents that are present in the BE extract. Thus, although the technical bases of these two detection methods are different, the strong correlation between the two methods is promising and should be further validated with application to a wider range of sediment BE concentrations and PAH mixture compositions.

Once the sediment BE method has been optimized through standardization efforts, several extensions of this method are envisioned. Biomimetic extraction TUs can be incorporated into mixture toxicity models that account for other classes of sediment stressors (e.g., ammonia, sulfide, metals) as a chemical line of evidence for sediment site characterization. This chemistry line of evidence can be compared with other lines of evidence, such as sediment toxicity testing or community impacts based on benthic health field surveys (Tuikka et al., 2011: Wang et al., 2021). Sediment BE measurements can also be more routinely incorporated into the design of sediment toxicity tests so that species/endpoint-specific BE threshold values can be derived. These BE toxicity thresholds can then be used to refine FCV_pdms_ estimates presented in this study as well as support species-specific TU metrics as described in the previous section.

Another logical extension of this work is to integrate sediment BE measurements into evaluation of remedy effectiveness. This includes use of both laboratory and field studies performed at various stages of a remedy from preremediation feasibility testing to postremediation monitoring (Grundy et al., 2023). For example, rather than using individual PAH analyses as the basis for cap design or field monitoring of cap performance (Garza-Rubalcava et al., 2023; Shen et al., 2018), BE analyses could alternatively be used to quantify how different remedies reduce risks associated with the complex, bioavailable mixture of contaminants in site sediments. This ability to quantify the bioavailability of site-specific mixtures in selecting and evaluating remedies for contaminated sediments would provide an important step advance to the current state of practice.

## Supplementary Material

vjae008_Supplementary_Data

## Data Availability

All data used in this study are included in the supplementary information online.
