# Supplementary material for: Integrating ex situ biomimetic extraction analyses into contaminated sediment assessment and management decisions
Source: Integr Environ Assess Manag. 2025 Jan 6;21(1):195–207. doi: 10.1093/inteam/vjae008 (PMC11804877; doi:10.1093/inteam/vjae008)
Supplement: vjae008_Supplementary_Data [file vjae008_supplementary_data.zip › Supplemental Figures Final.docx]

**SUPPLEMENTAL FIGURES**


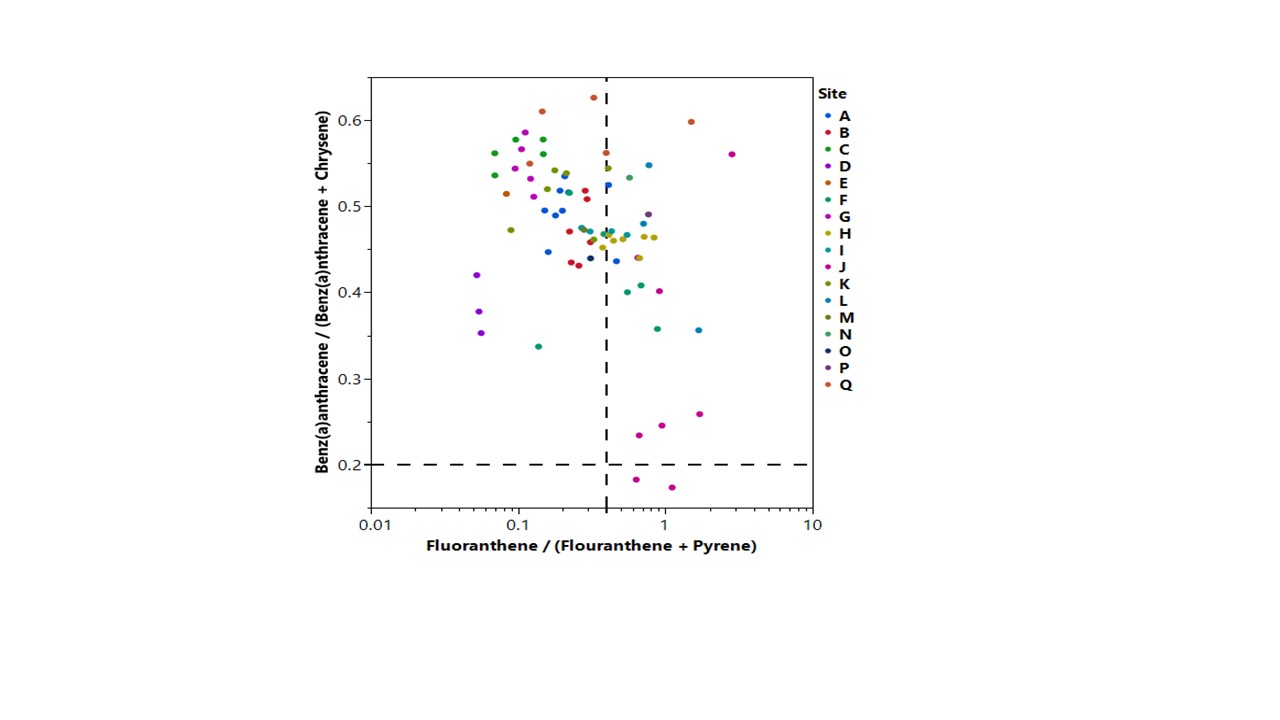


Figure S1. Double ratio diagnostic plot for evaluating PAH sources across study samples and sites. Ratios were constructed using data provided in Table S1.


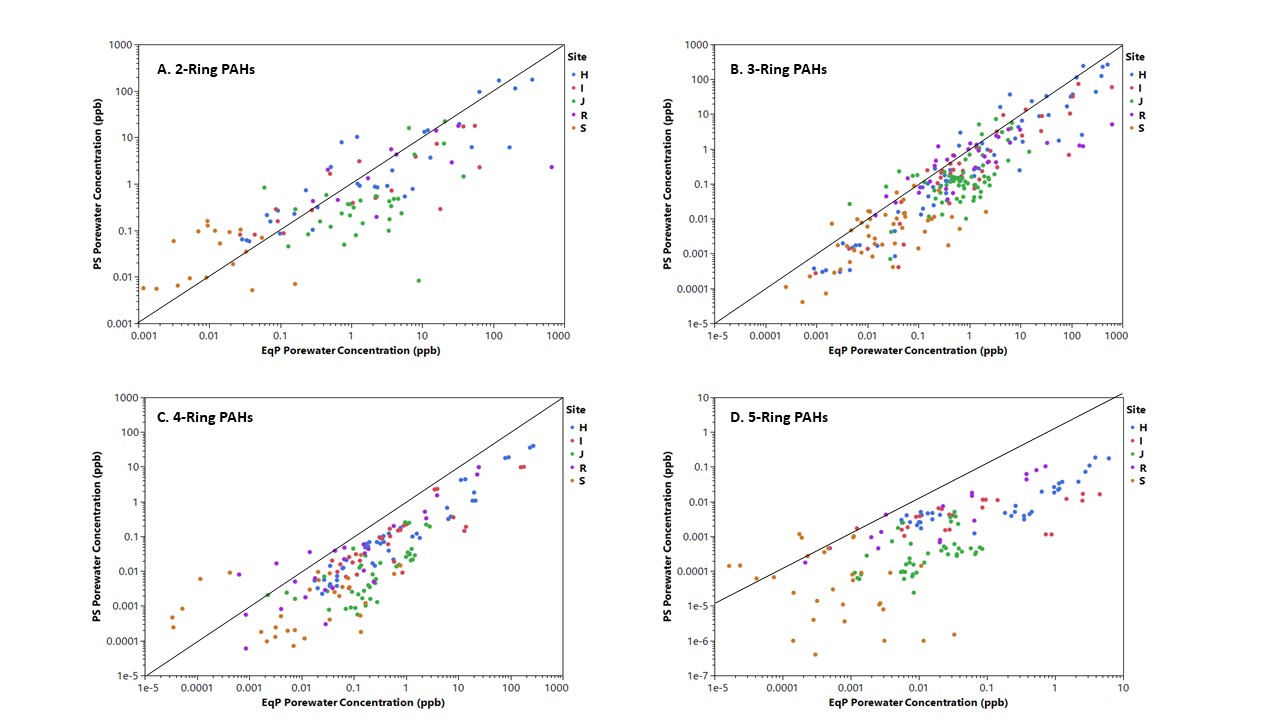


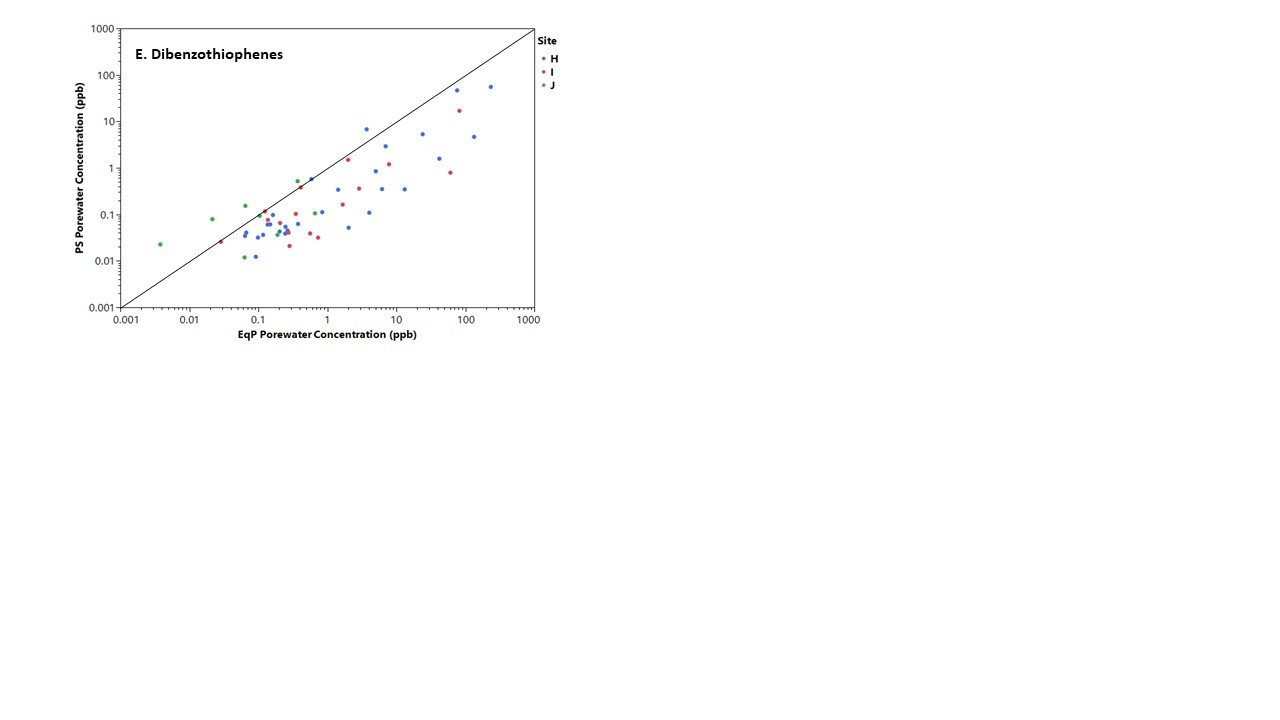


Figure S2. Comparison of measured versus predicted porewater concentrations derived using either *ex-situ* passive sampling (PS) measurements or equilibrium partitioning (EqP) predictions for individual target analytes from different hydrocarbon classes.


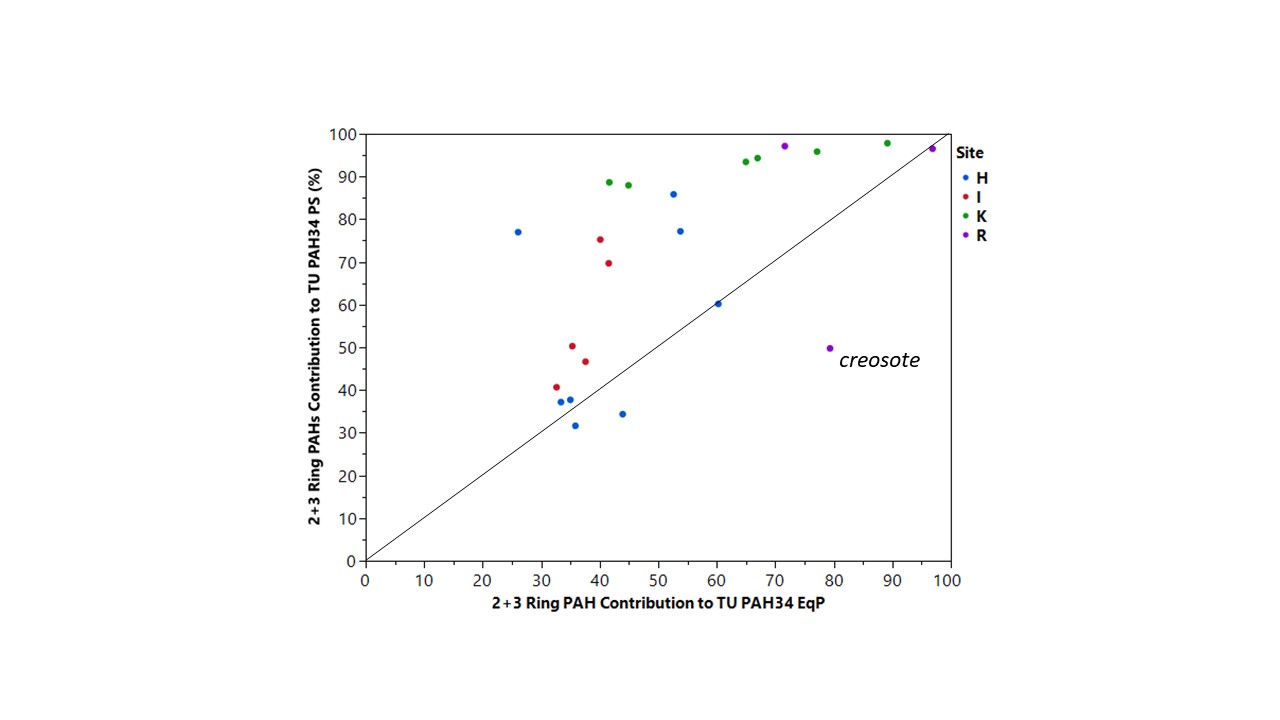


Figure S3. Contribution of 2 + 3 ring PAH classes to TU PAH34 derived using passive sampling (PS) measurements or equilibrium partitioning (EqP) predictions.


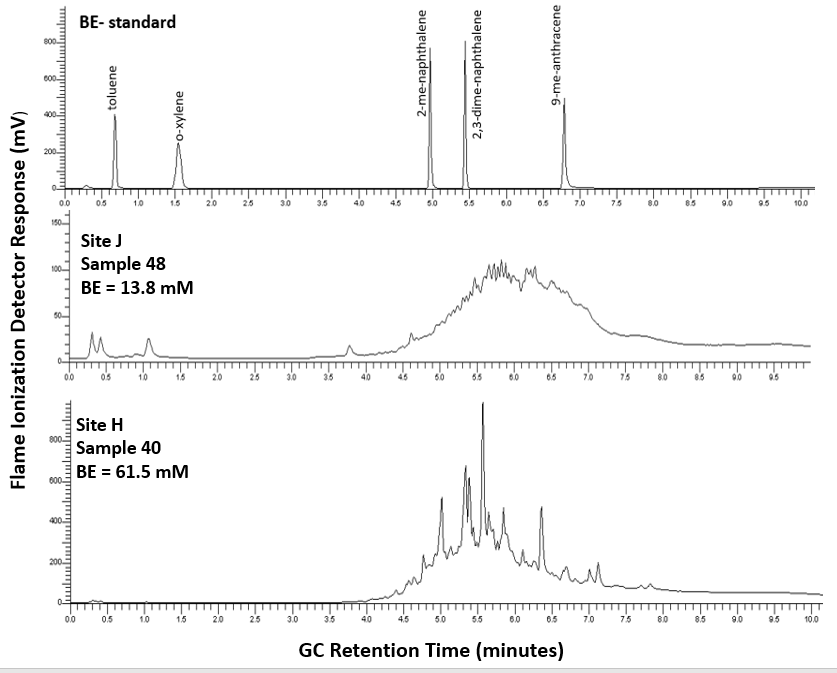


Figure S4. GC-FID chromatograms of the BE standard and two illustrative sediment samples evaluated using ex-situ passive sampling with polydimethylsiloxane coated fibers.

Figure S5. GC-FID detector response for calibration with 2,3-dimethyl naphthalene. Subsequent QC injections of standards performed between analysis of sediment BE extractions confirm detector stability following calibration.

`


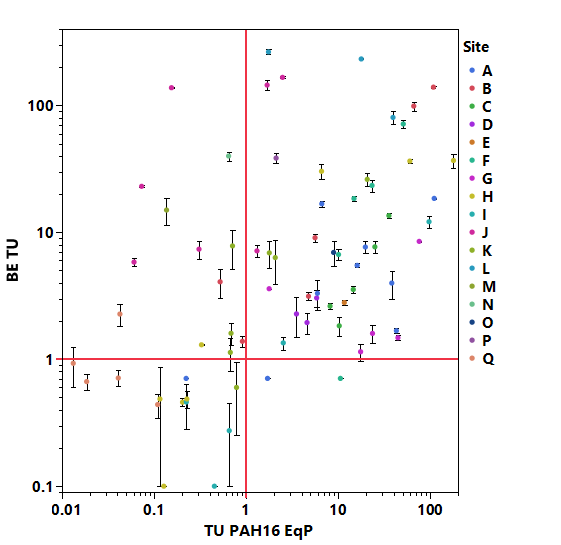


Figure S6. Correlation of Toxic Units derived from ex-situ BE sediment analysis with predictions from equilibrium partitioning based on PAH16.


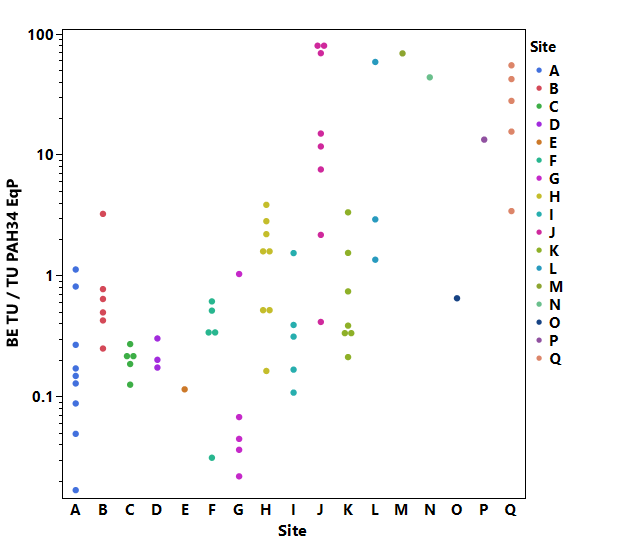


Figure S7. Ratio of Toxic Units calculated using BE measurements to Toxic Units derived using EqP calculations based on PAH34 for sediment samples collected across different sites. TU derived from EqP for site Q is based only on 25 PAHs that were reported for this study.


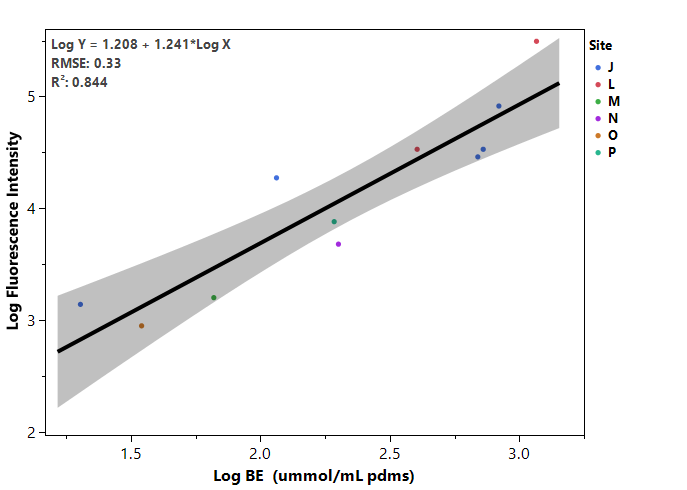


Figure S8. Correlation between fluorescence intensity of BE extracts and sediment BE concentrations determined using GC-FID for a subset of eleven sediments collected from six different sites.
